# Assessment of Knowledge and Attitude of Antenatal Mothers Regarding Physical Activity During Pregnancy: A Cross-Sectional Analytical Study at a Tertiary Care Hospital in Puducherry, India

**DOI:** 10.7759/cureus.72465

**Published:** 2024-10-27

**Authors:** Jebisha J, Sivasankari K, Parvathi T

**Affiliations:** 1 Department of Obstetrics and Gynaecology Nursing, Jawaharlal Institute of Postgraduate Medical Education and Research, Puducherry, IND; 2 Department of Obstetrics and Gynaecology, Jawaharlal Institute of Postgraduate Medical Education and Research, Puducherry, IND

**Keywords:** antenatal exercise, antenatal mothers, attitude, knowledge, physical activity

## Abstract

Introduction: A woman's body experiences numerous physiological and psychological changes throughout pregnancy in order to accommodate the expanding requirements of the growing fetus. These changes can lead to various pregnancy-related health issues like gestational diabetes, and pre-eclampsia. Women who regularly engage in physical activity during pregnancy have a reduced risk of developing those health problems. Pregnant women should have adequate knowledge and a good attitude towards physical activity during pregnancy, which is an essential precursor for practising exercise. Therefore, this study aimed to assess the knowledge and attitude of antenatal mothers regarding physical activity during pregnancy at a tertiary care hospital in Puducherry.

Method: A cross-sectional analytical study design was performed to collect data from 380 antenatal mothers. A convenient sampling technique was used to select the study participants. A structured, validated, self-administered questionnaire was used to collect data from pregnant women attending the antenatal outpatient department of Jawaharlal Institute of Postgraduate Medical Education and Research (JIPMER). Data were coded and analysed by IBM SPSS Statistics for Windows, Version 19.0 (Released 2010; IBM Corp., Armonk, New York, United States).

Results: This study revealed that almost half of the antenatal mothers (n=195, 51.3%) had adequate knowledge whereas 185 (48.7%) had inadequate knowledge regarding physical activity during pregnancy. Regarding their attitude, most of the antenatal mothers (n=303, 79.7%) had a favourable attitude and 77 (20.3%) had an unfavourable attitude towards physical activity during pregnancy. It was found that there is a significant association between education, employment, pre-pregnancy physical activity status, co-morbidities, and BMI of the antenatal mothers and their level of knowledge (p < 0.05). Also, educational status, employment status, level of activity in day-to-day life, pre-pregnancy physical activity status, co-morbidities, and number of antenatal visits attended by the antenatal mothers were significantly associated with their level of attitude (p < 0.05).

Conclusion: The level of knowledge regarding physical activity during pregnancy was adequate among antenatal mothers and their attitude seems to be favorable. This does not mean that they are exercising effectively. Therefore, health professionals should put forth their effort to bring about behavioural change among pregnant women towards exercise by conducting counselling sessions, and antenatal exercise classes during their antenatal care visits in order to empower pregnant women to lead a healthy pregnancy.

## Introduction

Many physiological and psychological changes in a woman's body occur throughout pregnancy to meet the expanding demands of the developing fetus [1,2]. A lot of pregnancy-related health problems, such as low back pain, hypertension, gestational diabetes mellitus (GDM), pre-eclampsia, intrauterine growth restriction, urinary incontinence, mental health disorders, and maternal obesity, might result from these changes [2].

Any movement of the body that arises from the skeletal muscle contraction can be considered physical activity and can occur at any time during life. Exercise, on the other hand, is a particular kind of physical activity where the skeletal muscles contract in a planned, controlled, and repetitive manner [3]. Women who regularly engage in physical activity during pregnancy have a lower risk of developing the above-mentioned health problems [2].

As per the recommendations of the World Health Organization (WHO) and the American College of Obstetricians and Gynecologists (ACOG), all pregnant women should be involved in moderate-intensity physical activity for a minimum of 150 minutes per week or 20-30 minutes per day on most or all days of the week during pregnancy [4,5].

According to the National Health and Nutrition Examination Survey (NHANES) 1999-2006, in the United States, only 13.8-22.9% of pregnant women met the physical activity recommendations [6]. Physical inactivity has been recognized by the WHO as the fourth most important risk factor for general population death worldwide [7]. According to ACOG standards estimation, nearly 79% of pregnant women attain a sedentary life [8]. Based on recent research, the percentage of pregnant women who were not physically active ranged from 27.2% to 88.9% [9].

Studies have reported that numerous benefits can be gained from physical activity during pregnancy, such as a decreased chance of pre-eclampsia, a decreased risk of gestational diabetes, overweight, preterm births, lower caesarean section, postpartum healing period, labour and delivery duration, and a reduction in lower back pain. Additionally, exercise during pregnancy promotes better sleep, alleviates tiredness, tension, anxiety, and depression, and enhances the health of the growing fetus and the mother [10].

Only 35.2% of pregnant women in India [11], 29% in Campinas, Brazil [12], 13.6% in Colombo, Sri Lanka [13], 20% in Pakistan [14], 27.2% in Serbia [15], and 37.9% in Gondar town, Northern Ethiopia [16], actually exercised during their pregnancy [3]. Globally, women's knowledge of physical exercise during pregnancy was estimated at only 19% in Zambia [17], 46.6% in Nigeria [18], and 39.5% in Northwest Ethiopia [8].

There are numerous factors that affect physical activity habits in pregnant women including low maternal education, unemployment, pregnancy symptoms or discomforts, lack of time, lack of motivation, safety concerns regarding their growing fetus, absence of social assistance, lack of access to resources and facilities, cultural and religious beliefs and adverse weather conditions [10].

The attitude of pregnant women toward physical activity also affects their practice during pregnancy. Studies showed that most pregnant women have poor attitudes towards physical activity during pregnancy. In Pakistan, 87.2% [19], and in Northwest Ethiopia, 55.3% of the pregnant women had negative attitudes [8].

The advantages of regular physical activity for an uncomplicated pregnancy greatly exceed the hazards to the pregnant woman and her developing fetus [5]. Physical activity during pregnancy seems to get little attention despite its advantages and affordability, and a majority of pregnant women choose to remain sedentary [20]. Sufficient understanding and a positive outlook on physical activity during pregnancy are thus important [8]. Pregnant women who don't know enough about exercise or who are afraid of the risks associated with it end up quitting or refusing to exercise [21].

A thorough understanding of current knowledge and attitudes regarding physical activity among antenatal mothers is necessary for developing holistic maternal health policies to address every aspect of risk. Therefore, this study intended to assess the knowledge and attitude regarding physical activity during pregnancy among antenatal mothers and to identify the factors associated with their level of knowledge and attitude.

## Materials and methods

This was a cross-sectional analytical study carried out to assess the knowledge and attitude regarding physical activity during pregnancy among antenatal mothers attending the antenatal outpatient department at Jawaharlal Institute of Postgraduate Medical Education and Research (JIPMER), Puducherry, India, between September 1, 2023, to October 21, 2023. The study was approved by the Nursing Research Monitoring Committee, JIPMER (approval number: JIP/CON/NRMC/M.Sc./2022/OBG/2) and the Institutional Ethics Committee of JIPMER (approval number: JIP/CON/IEC/M.Sc./2022/OBG/2). Confidentiality of the data, the right to withdraw from the study, and the subjects' anonymity were explained before data collection and an informed written consent was obtained.

Inclusion and exclusion criteria

Antenatal mothers who were at 28-36 weeks of gestation, primigravida, with singleton pregnancy, and who could understand, read, or write Tamil and/or English were included in the study using a consecutive sampling technique. Antenatal mothers who were already enrolled in exercise programs, with bad obstetrical history, placenta previa, gestational hypertension and gestational diabetes were excluded.

Sample size calculation

The sample size was estimated to be 380 by anticipating a proportion of favourable attitudes about physical activity during pregnancy among antenatal mothers as 55% based on a study conducted by Janakiraman et al. [16] and an absolute precision of 5% at a 5% level of significance.

Study tool

The knowledge and attitude of antenatal mothers were evaluated by administering a structured and validated knowledge and attitude questionnaire (see Appendices) which consisted of three sections. Section I included sociodemographic characteristics like age, marital status, educational status, employment status, activity level, income, residence, habit of doing exercise and obstetrical characteristics like gestational age, height, weight, history of abortion, comorbid illnesses, and number of antenatal visits. Section II included 11 structured knowledge questions regarding physical activity during pregnancy. Each correct answer carried one mark with a total score of 11, and the knowledge score was categorised into adequate knowledge (>50%) and inadequate knowledge (<50%). Section III included 15 structured statements regarding physical activity during pregnancy. Out of 15, seven were negative statements. A five-point Likert scale was used with opinions ranging from strongly agree to strongly disagree. The positive statements were scored as four, three, two, one, zero, and for the negative statements, reverse scoring was done. According to the total score obtained, attitude towards physical activity during pregnancy among antenatal mothers was categorised into favourable attitude (>50%) and unfavourable attitude (<50%).

Data analysis

Data were analysed using the IBM SPSS Statistics for Windows, Version 19.0 (Released 2010; IBM Corp., Armonk, New York, United States). The categorical variables such as educational status, employment status, residence, marital status, history of abortion, habit of doing exercise, and level of knowledge and attitude were expressed as frequency and percentage. The continuous variables such as age, income, height, weight, gestational age, and number of antenatal visits were expressed as median with interquartile range (IQR) according to the distribution of data. The association of all the categorical variables with the level of knowledge and attitude was assessed using the Chi-square test or Fisher’s exact test. The comparison of all the continuous variables between the level of knowledge and attitude was analysed using the Mann-Whitney U test. All the statistical analysis was carried out at a 5% significance level, and p < 0.05 was considered significant.

## Results

A total of 380 antenatal mothers were included in the study. The distribution of categorical variables according to sociodemographic and obstetrical characteristics is described in Table 1. Additionally, the median (IQR) of age, monthly income, gestational age and BMI were 25 (22, 27), 10000 (5000, 15000), 32 (30, 35) and 25.25 (22.42, 28.50), respectively.

**Table 1:** Distribution of categorical variables among antenatal mothers according to sociodemographic and obstetrical characteristics (N=380)

Variables	Categories	Frequency	Percentage
Marital status	Married	378	99.5
	Divorced	2	0.5
Education	Primary education	16	4.2
	Secondary education	110	28.9
	Graduate and above	253	66.6
	Non-formal education	1	0.3
Employment	Employed	54	14.2
	Unemployed	326	85.8
Activity level	Sedentary	31	8.2
	Moderate	336	88.4
	Heavy	13	3.4
Pre-pregnancy Physical Activity	Yes	113	29.7
	No	267	70.3
History of abortion	Yes	32	8.4
	No	348	91.6
Co-morbidity	Yes	27	7.1
	No	353	92.9
Number of antenatal visits	One	137	36.1
	Two	78	20.5
	Three and above	165	43.4

Knowledge regarding physical activity during pregnancy

**Figure 1:**
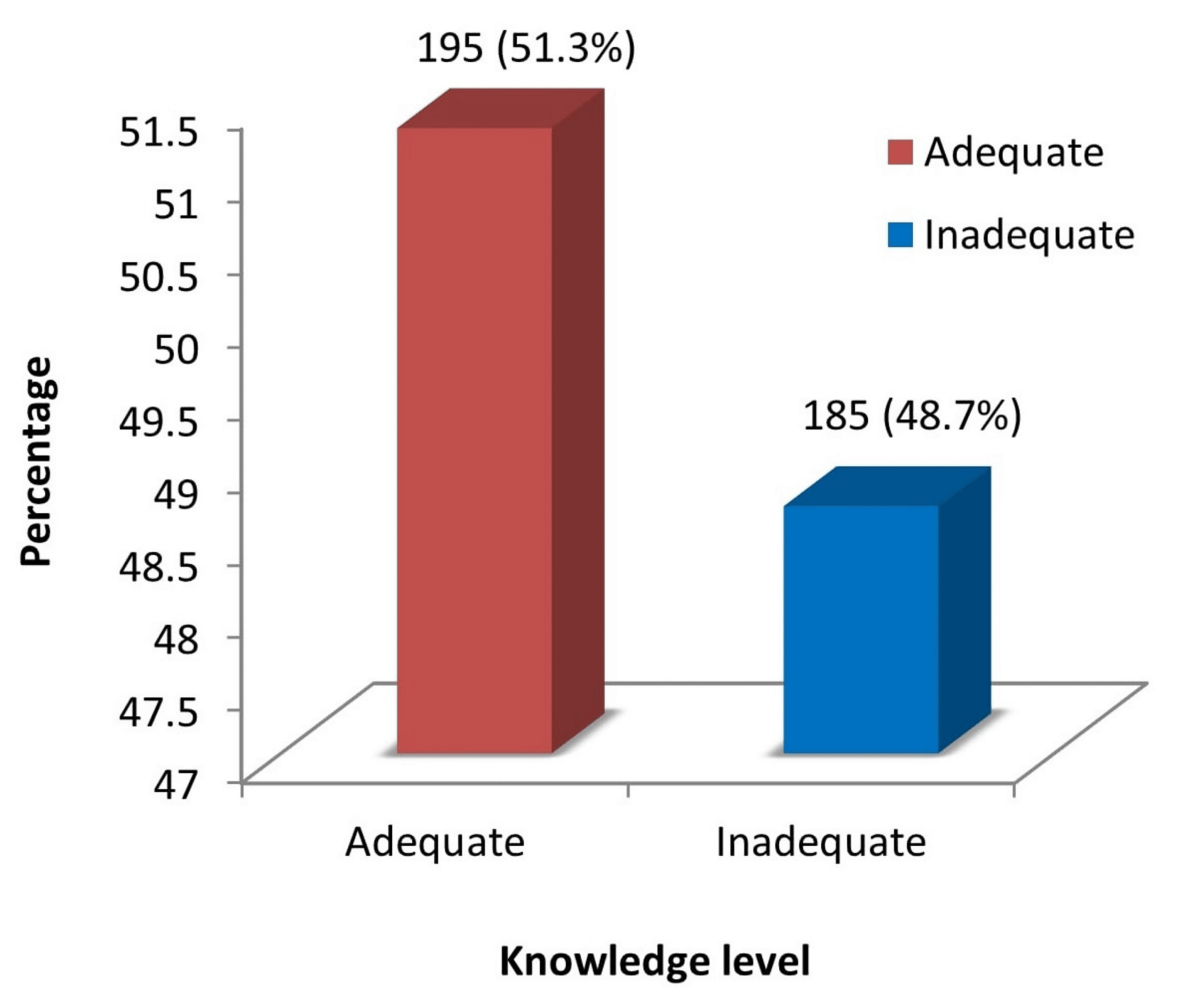
Level of knowledge regarding physical activity during pregnancy among antenatal mothers (N=380)

**Figure 2:**
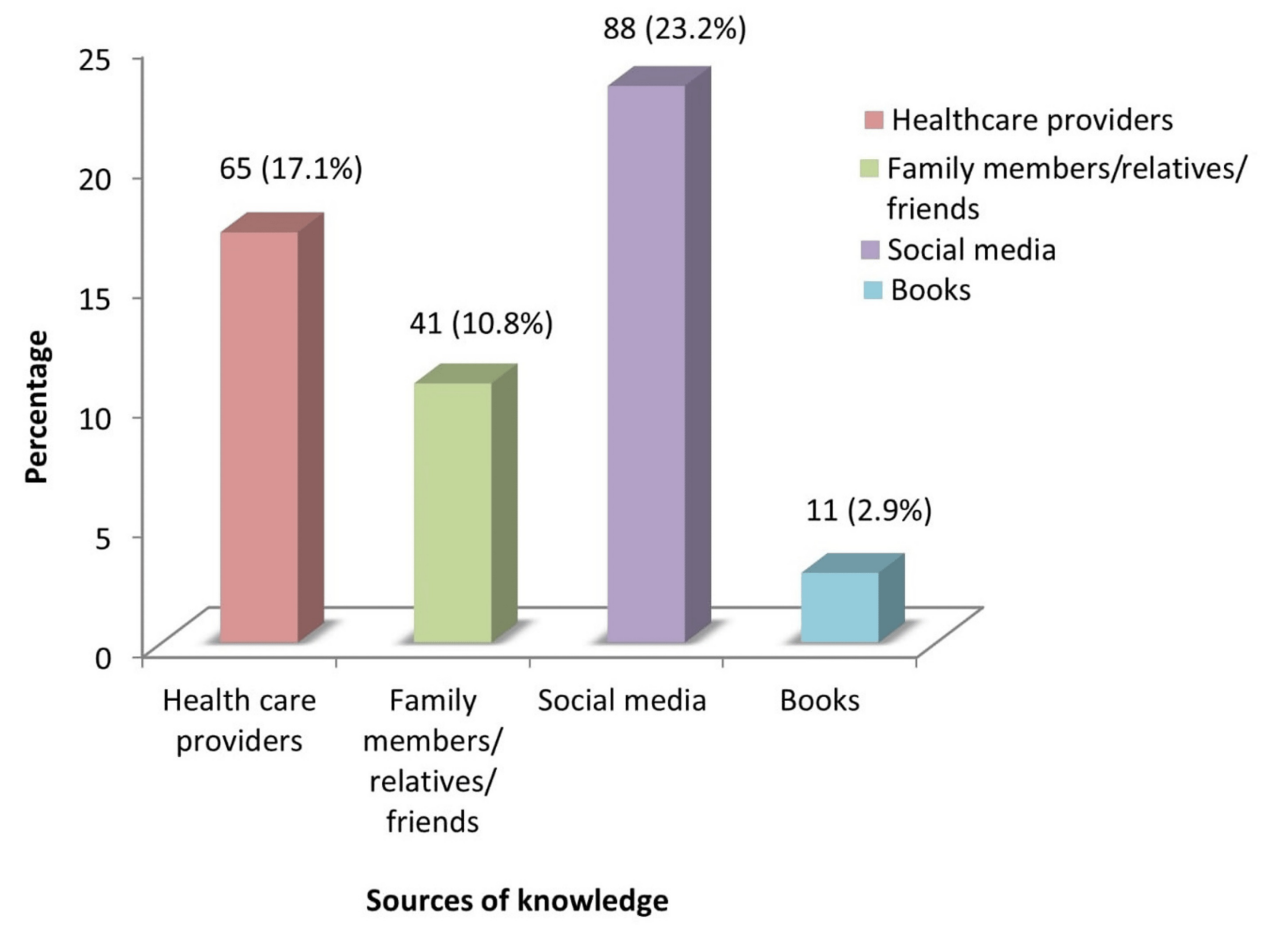
Sources of knowledge regarding physical activity during pregnancy A total of 205 participants of the 380 participants responded to this. Percentages were calculated on the basis of total number of participants (N=308) Sources were given based on their opinion and we categorized them as healthcare workers, family/friends/relatives, social media (in which we included all sources related to WhatsApp, Instagram, reels/shorts, Internet, etc), and books (in which we included all print materials such as journals/newspapers etc).

With respect to the level of knowledge regarding physical activity during pregnancy, 51.3% had adequate knowledge and 48.7% had inadequate knowledge (Figure 1). Figure 2 demonstrates the sources of knowledge regarding physical activity during pregnancy among antenatal mothers.

Table 2 shows that knowledge regarding physical activity during pregnancy among antenatal mothers had a significant association between educational status (p=2446E05), employment status (p = 0.006), pre-pregnancy physical activity status (p=5242E05), and co-morbidities (p = 0.040). However, there was no significant association between marital status, activity level in day-to-day life, history of abortion, and number of antenatal visits attended. In addition to the information provided in Table 2, it was found that the antenatal mothers' level of awareness about physical activity during pregnancy was significantly associated with their BMI (p = 0.006). However, there was no significant association between the variables like age, monthly income of the family, and gestational age.

**Table 2:** Association of the level of knowledge regarding physical activity during pregnancy with sociodemographic and obstetrical variables of the participants (N=380) ^*^p<0.05 (significant), ^#^Fisher’s exact test

Variables	Level of knowledge				Chi-square χ2/ Fisher’s exact test	p-value
	Inadequate (n = 185)		Adequate (n = 195)			
	Frequency	Percentage	Frequency	Percentage		
Marital status					-#	0.236
Married	183	48.4	195	51.6		
Divorced	2	100.0	0	0.0		
Educational status					21.755^#^	2446E05*
Primary education	10	62.5	6	37.5		
Secondary education	72	65.5	38	34.5		
Graduate and above	102	40.3	151	59.7		
Non-formal education	1	100.0	0	0.0		
Current employment status					7.456	0.006*
Employed	17	31.5	37	68.5		
Unemployed	168	51.5	158	48.5		
Level of activity in day-to-day life					0.275	0.872
Sedentary	16	51.6	15	48.4		
Moderate	162	48.2	174	51.8		
Heavy	7	53.8	6	46.2		
Pre-pregnancy physical activity status					16.358	5242E05*
Yes	37	32.7	76	67.3		
No	148	55.4	119	44.6		

Attitude regarding physical activity during pregnancy

**Figure 3:**
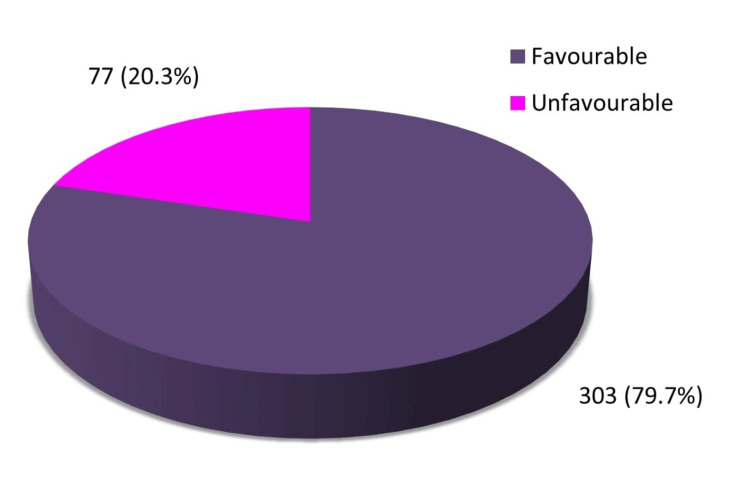
Level of attitude regarding physical activity during pregnancy among antenatal mothers (N=380)

With respect to the attitude regarding physical activity during pregnancy among antenatal mothers, 79.7% had a favourable attitude while 20.3% had an unfavourable attitude (Figure 3).

Table 3 describes that attitude regarding physical activity during pregnancy among antenatal mothers has a significant association between educational status (p=4712E06), employment status (p = 0.011), level of activity in day-to-day life (p = 0.039, pre-pregnancy physical activity status (p = 0.006), co-morbidities (p = 0.007) and number of antenatal visits attended (p = 0.001). However, there is no significant association between the variables like marital status and history of abortion. Also, there is no significant association between the level of attitude among antenatal mothers regarding physical activity during pregnancy and variables like age, gestational age, family monthly income, and BMI.

**Table 3:** Association of the level of attitude regarding physical activity during pregnancy with sociodemographic and obstetrical variables of participants (N=380) ^*^ p<0.05 (significant), ^#^ Fisher’s exact test

Variables	Level of attitude				Chi-square χ^2^/ Fisher’s exact test	p-value
	Unfavourable (n = 77)		Favourable (n = 303)			
	Frequency	Percentage	Frequency	Percentage		
Marital status					-^#^	0.365
Married	76	20.1	302	79.9		
Divorced	1	50.0	1	50.0		
Educational status					25.127^#^	4712E06*
Primary education	7	43.8	9	56.3		
Secondary education	37	33.6	73	66.4		
Graduate and above	33	13.0	220	87.0		
Non-formal education	0	0.0	1	100.0		
Current employment status					6.439	0.011*
Employed	4	7.4	50	92.6		
Unemployed	73	22.4	253	77.6		
Level of activity in day-to-day life					6.087^#^	0.039*
Sedentary	10	32.3	21	67.7		
Moderate	62	18.5	274	81.5		
Heavy	5	38.5	8	61.5		
Pre-pregnancy physical activity status					7.636	0.006*
Yes	13	11.5	100	88.5		
No	64	24.0	203	76.0		
History of abortion					0.465	0.495
Yes	5	15.6	27	84.4		
No	72	20.7	276	79.3		
Co-morbid illness					7.386	0.007*
Yes	0	0.0	27	100.0		
No	77	21.8	276	78.2		
Number of antenatal visits attended					13.282	0.001*
One	15	10.9	122	89.1		
Two	16	20.5	62	79.5		
Three and above	46	27.9	119	72.1		

## Discussion

This study reveals that almost half of the antenatal mothers (n=195, 51.3%) had adequate knowledge and 185 (48.7%) had inadequate knowledge about physical activity during pregnancy. These findings are congruent with a study conducted by Sitot and Workye [22]. Their study demonstrated that out of 255 pregnant mothers, 51% (n=130) had good knowledge about antenatal exercise. Similarly, another study conducted by Jahan and Anaiba concluded that 56.1% of pregnant women demonstrated good knowledge, while 43.9% had poor knowledge regarding antenatal exercise [23]. Likewise, the study conducted by Asante et al. with 77 participants revealed that 57 (74.0%) had a high level of knowledge, 14 (18.2%) had moderate knowledge, and six (7.8%) had a low level of knowledge regarding physical activity [24].

It is clearly observed from the present study that 23.2% of antenatal mothers obtained information about antenatal exercise from social media, 17.1% from healthcare providers, 10.8% from family members/relatives/friends and very few of them (2.9%) from books. Similar results were found in the study by Szatko et al., which revealed that 50% of the participants) got their information on physical activity during pregnancy from the internet, 32.3% from books, 22.4% from doctors, and 18.9% from midwives [5].

Hence, it can be interpreted that antenatal mothers in the present study had adequate knowledge regarding physical activity during pregnancy. Also, it was found that their main source of knowledge was social media. This highlights the need for the provision of antenatal exercise classes by doctors and midwives for the mothers during their antenatal visits which would help them to address their doubts and further increase their knowledge regarding physical activity during pregnancy.

Also, this study showed that most of the antenatal mothers (n=303, 79.7%) had favourable attitudes. These results are consistent with the study conducted by Benyian among 200 pregnant women to evaluate their knowledge and attitude towards physical exercise during pregnancy which showed that most (73%) had a positive attitude towards physical exercise during pregnancy [25]. The findings of the present study were also supported by that of Negash and Alelgn's study in which most participants (63.7%) had a positive attitude towards antenatal exercise [8]. The results of the study conducted by Janakiraman et al. revealed that more than half of the pregnant women (55.3%) had a favourable attitude towards antenatal exercises [16].

In the present study, select sociodemographic variables like educational status, employment status, pre-pregnancy physical activity status, co-morbidities, and BMI of the antenatal mothers had a significant association with their level of knowledge (p < 0.05). The results of this study also demonstrate a significant association (p < 0.05) between their attitude and variables like number of prenatal visits attended, employment status, level of activity in daily life, pre-pregnancy physical activity status, and educational status. Other sociodemographic variables like marital status, age, gestational age, monthly income, history of abortion, and BMI did not show a significant association with the level of attitude.

These findings are congruent with a study conducted by Gari et al. in which the level of beliefs, awareness, and knowledge regarding antenatal exercise was significantly associated (p < 0.05) with age, marital status, education level, occupation, and number of living children; however, the level of beliefs, awareness, and knowledge regarding antenatal exercise was not found to be significantly associated (p = 0.640) with region and income (p = 0.298) [26]. A similar study conducted by Bayisa et al. revealed a significant association (p < 0.05) between the women's knowledge level, education, exercise performed before pregnancy, and whether they had ever heard of exercise during pregnancy [7]. Also, their study revealed that pregnant women who were knowledgeable or whoever heard of exercise during pregnancy had shown a significant association with the level of attitude (p < 0.05).

Limitations and strengths

This study had certain limitations as only primigravida mothers between 28-36 weeks of gestation with singleton pregnancy were the samples. The data collection period for this study was limited to six to eight weeks and the study was conducted only in JIPMER OPD settings. Despite drawbacks, this study assessed the knowledge and attitude of antenatal mothers regarding physical activity during pregnancy, which is not yet well explored in India. Generalization of the study findings can be made to the target population because of the large sample size. The strength of the study was that there was a 100% response rate, which was probably because the data collection at the antenatal OPD setup was convenient for the participants.

## Conclusions

This study shows that almost half of the antenatal mothers had adequate knowledge and attitude regarding physical activity during pregnancy. However, this doesn’t necessarily imply that they were practising exercise well. Thus, more efforts should be undertaken by health experts to increase awareness of the importance of physical activity during pregnancy, thereby emphasizing behavioural change among antenatal mothers towards antenatal exercise. Furthermore, counselling sessions and antenatal exercise classes should be conducted during the antenatal care visits in order to empower antenatal mothers to lead a healthy pregnancy.

## Figures and Tables

**Figure 4:**
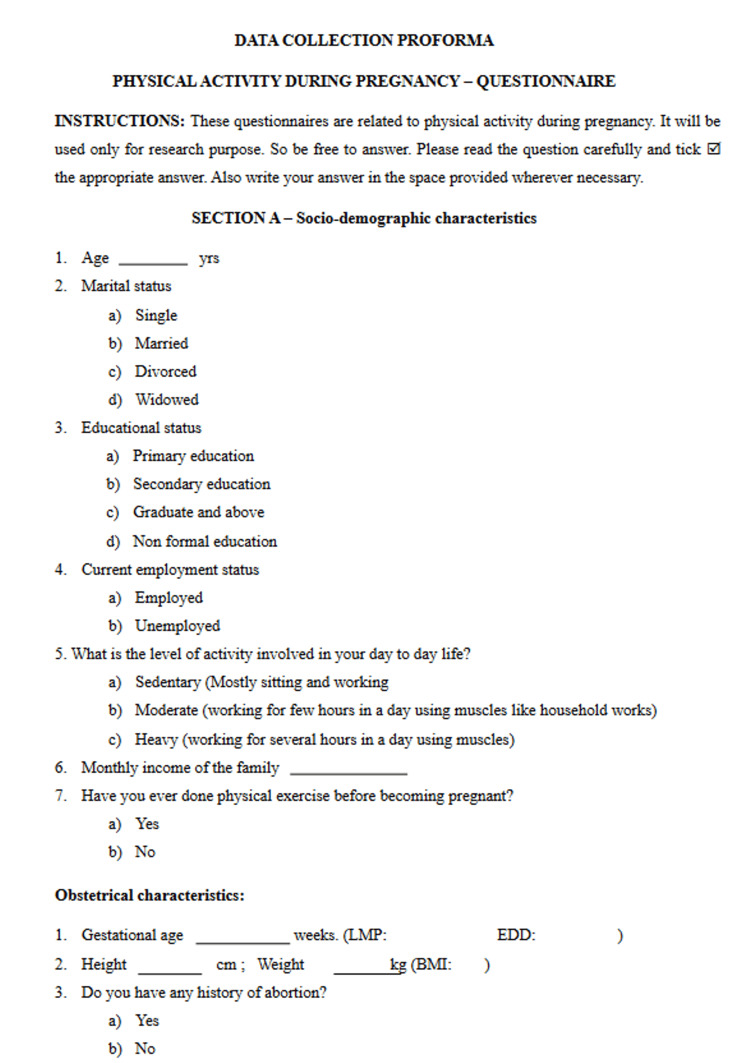
Questionnaire, page 1

**Figure 5:**
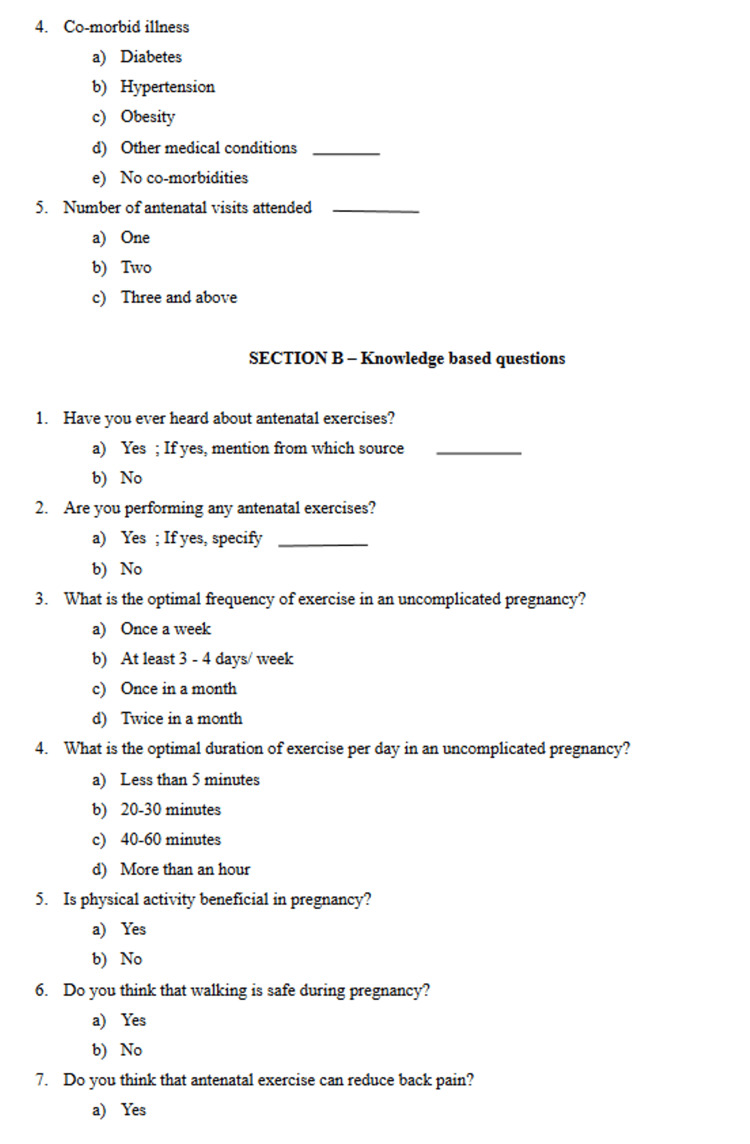
Questionnaire, page 2

**Figure 6:**
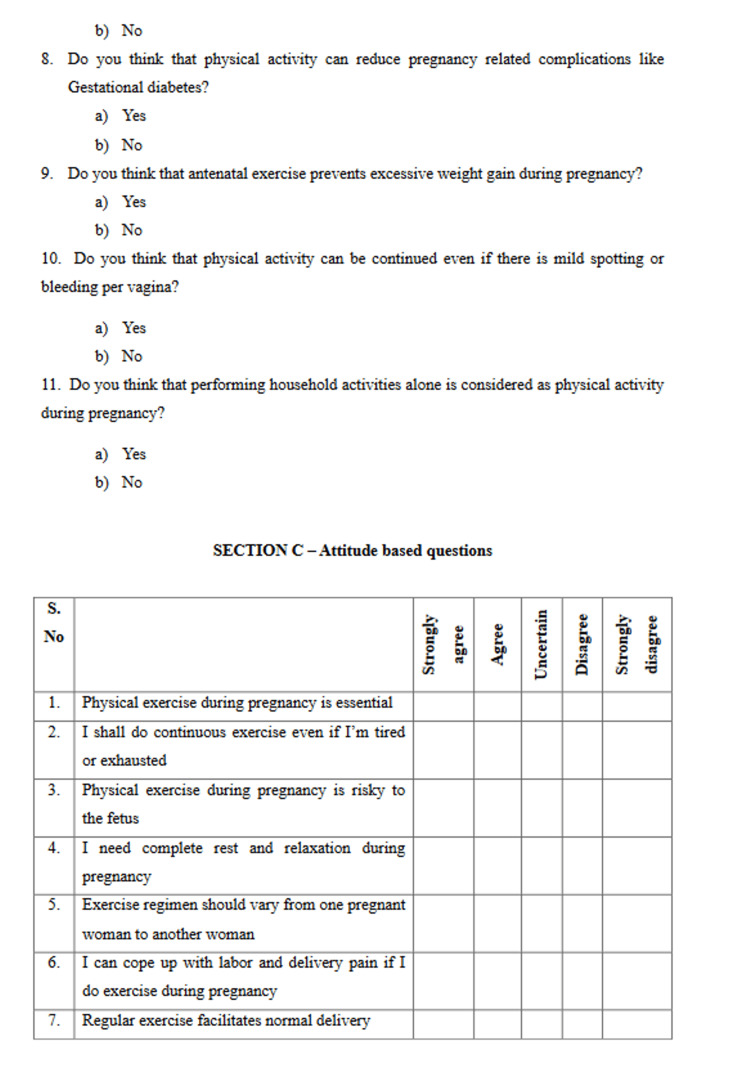
Questionnaire, page 3

**Figure 7:**
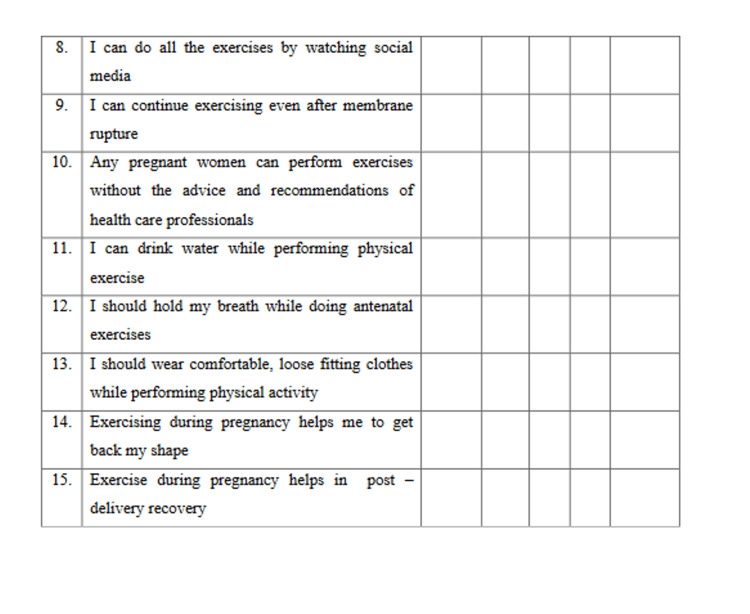
Questionnaire, page 4

## Appendices

Questionnaire
